# Depressive Symptoms Affect Cognitive Functioning from Middle to Late Adulthood: Ethnoracial Minorities Experience Greater Repercussions

**DOI:** 10.1007/s40615-024-02121-x

**Published:** 2024-08-15

**Authors:** Michael J. Persin, Ameanté Payen, James R. Bateman, Maria G. Alessi, Brittany C. Price, Jeanette M. Bennett

**Affiliations:** 1 Department of Psychological Science, UNC Charlotte, 9201 University City Blvd, 4018 Colvard, Charlotte, NC 28223 USA; 2 Health Psychology PhD Program, UNC Charlotte, Charlotte, USA; 3 Department of Neurology, Wake Forest University School of Medicine, Winston-Salem, USA; 4 Alzheimer’s Disease Research Center, Wake Forest University School of Medicine, Winston-Salem, USA

**Keywords:** Depressive symptoms, Midlife, Cognitive functioning, Ethnoracial differences

## Abstract

Cognitive deficits, a diagnostic criterion for depressive disorders, may precede or follow the development of depressive symptoms and major depressive disorder. However, an individual can report an increase in depressive symptoms without any change in cognitive functioning. While ethnoracial minority group differences exist, little is known to date about how the relationship between depressive symptoms and cognitive function may differ by ethnoracial minority status. Utilizing data from the Midlife in the United States (MIDUS) study waves II (M2) and III (M3), this study examines the relationship between depressive symptoms and cognitive functioning concurrently and longitudinally in community-dwelling adults, as well as whether the results differed by ethnoracial minority status. Our participants included 910 adults (43.8% male, 80.8% White, 54.4 ± 11.5 years old at M2). Cross-sectionally, depressive symptoms, ethnoracial minority status, and their interaction had significant effects on cognitive function, consistent with previous investigations. Longitudinally, higher M2 depressive symptoms predicted poorer cognitive function at M3 over and above M2 cognitive functioning, but only within the ethnoracial minority sample. Our finding suggests that depressive symptoms predict cognitive functioning both concurrently and across time, and this relationship is moderated by ethnoracial identity, resulting in greater cognitive deficits among ethnoracial minority groups compared to their non-Hispanic White counterparts.

Depression is a mood disorder that can occur at any age and typically classified as mild, moderate, or severe [1]. In adults, depression is defined by the presence of depressed mood and/or reduced interest or pleasure in most activities [anhedonia] and is often accompanied by changes in cognition that impede the ability to think or concentrate, impacting day-to-day functioning [2]. According to the World Health Organization (WHO), the depression burden worldwide is estimated to be 280 million cases, or ~ 5% of the global population and nearly 6% of those over 65 years old [3]. Data from the US Department of Health and Human Services report that the age groups 45–64 and 65 and over are tied for the second highest prevalence of depression at 18.4%, just behind the 18–29 age group [4]. This is particularly concerning when considering symptom severity,moderate and severe depression rates in the 45–64 age group are 4.5% and 3.1%, respectively [4]. Furthermore, depression is estimated to predict a 20% increase in the risk of dementia [5] and may account for 5–11% of Alzheimer’s disease cases [6].

Impaired cognition can include problems with cognitive functions such as executive control and attention [7]. Furthermore, cognitive impairment is a diagnostic criterion for major depressive disorder [8] and has been associated with greater vulnerability to developing depression and increased risk of symptom relapse [9]. An estimated 60% of individuals with depression display impaired cognition [10–12]. Moreover, persistent moderate to high and increasing depressive symptoms during adulthood appear to drive cognitive decline over time [13–15]. These data suggest that depression and cognitive impairments may bidirectionally increase the risk of one another. A major limitation in these cross-sectional and longitudinal studies is the limited ethnoracial diversity of their samples, as the majority of samples have been predominantly within White/Caucasian populations, resulting in questions regarding the universality of the relationship between depression and cognitive impairment and whether the association is variable across different sociodemographic groups.

The prevalence rate of major depressive disorder is lower in African Americans than in their Caucasian counterparts at 10.4% and 17.9%, respectively [16, 17]. However, over 50% of cases among African Americans are chronic, lasting longer and reoccurring more frequently when compared to 39% in White individuals, suggesting that the burden of depression may fall heavier on the African American population and may result in more significant impairment across the functional spectrum [16]. Current prevalence rates may also underestimate the true incidence of depression in this population, as less than half of African Americans report seeking treatment for their disorder despite rating their depressive symptoms as severe and disabling [16, 18].

Similarly, the prevalence rate of major depression across other ethnoracial minorities is lower than in their Caucasian counterparts at 6.8% of Hispanics [19], and around 6.8% of Asian/ pacific islanders [20]. Another similarity is the disparity in treatment across these ethnoracial groups; specifically, among individuals with past year depressive episodes, over 50% of ethnoracial minorities did not access mental health treatment with Asians and Hispanics leading the way with 68.7% and 63.7%, respectively, compared to around 40% in the non-Hispanic Caucasian group [21]. Among individuals with severe subtype depression, ethnoracial minorities showed significantly lower rates of mental health service compared with Caucasians [22].

Consequently, these ethnoracial disparities in seeking mental health treatment result in different rates of antidepressant use [23–25]. Antidepressant use can have a positive effect on cognition functioning among individuals who have depression, while little to no effect has been observed on non-depressed populations [26]. However, antidepressants have also been shown to be of little use in combating the cognitive symptoms of depression such as poor concentration [27]. Given the wide use of antidepressants for depressive symptoms and other “off-label” conditions [28–30], examining the relationship between depressive symptoms and cognition function within this community-dwelling sample will be inclusive, regardless of antidepressant use.

In general, cognition declines with age [31]. While ethnoracial disparities in cognitive functioning are often reliably found, these disparities are not due ability and more likely explained by the cognitive measurement tools biasing European/Caucasian culture [32]. Furthermore, these well-accepted limitations of traditional cognitive screenings are evidenced in cultural and linguistic differences among groups leading to paradoxical declines among ethnic minorities and less consistency surrounding differences in cognitive decline over time [33, 34]. Beyond culturally biased assessment tools, sociodemographic factors like income, education, and occupation status also affect performance on cognitive tasks as these are all tied to greater opportunities throughout life and are associated with better health outcomes [35]. In the US, these sociodemographic factors often conflate or coincide with ethnoracial minority identity [36]. Specifically, ethnoracial minorities on average complete less education compared to their White counterparts and earn less income, even for the same job responsibilities [37–39].

This complex relationship is best encapsulated by the theory of cumulative advantage and disadvantage (CAD). CAD states that disadvantages lead to more disadvantages and adverse life events result in adverse health outcomes, while advantages lead to more advantages, creating a widening gap between the socioeconomic haves and have-nots [35]. Some researchers posit CAD as a possible explanation for ethnoracial disparities in cognition that may increase or widen the gap in cognitive performances between ethnoracial groups as they age [35]. This theory also relates to a physiological concept known as allostatic load that suggests the accumulation of physiological responses to stressful life events can lead to disruptions/dysregulation in or wear-and-tear of the biological systems, negatively altering overall health [40]. Higher allostatic load has been reliably linked to poorer cognition [41] and additional evidence suggests the elevated allostatic load may be related to neurostructural and neurofunctional alterations [42], thus, allostatic load, when available, should be included in analyses as a control variable when it is not the primary predictor.

Given CAD theory [35] and ethnoracial differences in cognitive functioning [32], we examine the effect of ethnoracial minority status on the relationship between depressive symptoms and cognitive functioning in middle-aged and older adults. Using publicly available data from the Midlife in the United States (MIDUS) study, this investigation provides insights into both the cross-sectional and longitudinal associations. We expect that depressive symptoms will significantly predict poorer cognitive functioning both concurrently and longitudinally. We also predict this relationship will be modified by ethnoracial minority status, such that ethnoracial minorities will exhibit greater decreases in cognitive functioning relative to their White counterparts. All analyses will be conducted with and without those individuals who reported using antidepressants at the second wave of data collection (M2) due to the known differential effects of antidepressants on cognitive functioning depending on depression diagnostic status.

## Method

### Study Data

This secondary data project utilized the publicly available Midlife in the United States (MIDUS) study waves two (M2) and three (M3), including the Milwaukee sub-study that consists of a Black/African American population. M2 data were collected from 2004 to 2009, while M3 data were collected from 2013 to 2017. At M2, data were aggregated across the main phone survey (*n* = 5555), cognitive phone testing (*n* = 4512), and biomarker study (*n* = 1255) datasets, yielding a total sample size of 1152 participants. At M3, only the cognitive dataset was utilized to investigate longitudinal cognitive outcomes. For more details about the MIDUS data collection methods, study designs, and participant attrition rates, please see the information reported elsewhere [43, 44].

### Participants

Participants (*n* = 242) were excluded from analyses due to the following criteria: incomplete M2 cognitive data (*n* = 136), incomplete depressive symptom data (*n* = 79), missing allostatic load covariate data (*n* = 25), and missing other covariate data (*n* = 2). Thus, the final M2 sample included 910 participants, 43.8% male, 80.8% White, and 54.4 ± 11.5 years old. At M2, 141 of the 910 participants, or 15.5%, endorsed being on an antidepressant. Due to attrition at M3, the longitudinal analyses included 741 participants, 42.2% male, 81.5% White, and 62.6 ± 10.6 years old. The ethnoracial minority group (*n* = 174) was comprised of those who identified as Black/African American (*n* = 129), Hispanic/Latino (*n* = 32), Native American/Alaskan Native (*n* = 6), Asian (*n* = 4), or Middle Eastern/North African (*n* = 3). The sample characteristics (mean and frequencies) of the study variables are represented in Table 1 for the overall group and separated by ethnoracial minority status.

**Table 1:** Summary descriptive statistics [mean (M) and standard deviation (SD) or frequency (*n*) and percentage (%)] of all study variables for the overall sample and by ethnoracial group status

Variables	Overall (*n* = 910)		Non-Hispanic White (*n* = 736)		All ethnoracial minorities (*n* = 174)	
	M/n	SD/%	M/n	SD/%	M/n	SD/%
M2 ethnoracial identity						
Non-Hispanic White	736	80.9%	736	100%	–	–
Black or African American	129	14.2%	–	–	129	74.1%
Hispanic/Latino	32	3.5%	–	–	32	18.4%
Native American/Alaskan native	6	0.7%	–	–	6	3.4%
Asian or Asian American	4	0.4%	–	–	4	2.3%
Middle Eastern/North African	3	0.3%	–	–	3	1.7%
M2 Sex						
Female	511	56.2%	396	53.9%	115^**^	66.1%
Male	399	43.8%	340	46.1%	59	33.9%
M2 body mass index (kg/m^2^)	29.7	6.5	29.2	5.9	31.9^†^	8.4
M2 education	7.6	2.5	7.7^***^	2.4	6.8	2.5
M2 total medications	5.8	4.6	6.0^**^	4.7	4.9	4.2
M2 antidepressant use (yes)	141	15.5%	124^*^	16.9%	17	9.7%
M2 allostatic load	8.8	3.2	8.8	3.3	9.0	2.9
M2 age (years)	54.4	11.5	54.8^**^	11.6	52.5	11.1
M3 age (years)^**a**^	62.6	10.6	63.2^†^	10.8	61.9	10.9
M2 depressive symptoms	8.9	7.9	8.5	7.7	10.7^***^	8.6
M2 BTACT z-scores						
Global cognition	0.13	0.92	0.25^***^	0.88	− 0.38	0.92
Executive functioning	0.18	0.90	0.30^***^	0.85	− 0.36	0.93
Episodic memory	0.08	0.91	0.11^*^	0.88	− 0.05	1.00
M3 BTACT z-scores						
Global cognition^**a**^	0.03	0.66	0.11^b***^	0.63	− 0.32^c^	0.68
Executive functioning^**a**^	− 0.10	0.71	− 0.02^b***^	0.67	− 0.48^c^	0.73
Episodic memory^**a**^	0.01	0.99	0.04^b^	0.97	− 0.10^c^	1.04

*n* = 910, unless otherwise noted. ^a^*n* = 741, ^b^*n* = 604, ^c^*n* = 137. *p* < .10, *p < .05, **p < *.01*, ***p < *.001.* Significant group differences indicated on the group with the higher value. *M2*, Midlife in the United States wave 2 data collection; *M3*, Midlife in the United States wave 3 data collection; *kg/m*^*2*^, kilograms/meter squared; *BTACT*, Brief Test of Adult Cognition by Telephone. For M2 education, 7 is the equivalent of an associate’s degree or 3 + years of college at a 4-year institution

### Measures

#### Depressive Symptoms

The well-validated Center of Epidemiologic Studies Depression (CES-D) scale, a self-report questionnaire primarily utilized in research settings, assessed current depressive symptoms at M2. A higher score indicates greater depressive symptoms. Individuals scoring between the range of 15–21 are suggestive of mild to moderate depression, and scores over 21 indicate likely major depression [45].

#### Cognitive Function

The Brief Test of Adult Cognition by Telephone (BTACT) was used to estimate global cognitive function. It was designed to assess cognitive differences in various cognitive domains, including episodic verbal memory, working memory, executive function, and processing speed of non-demented adults [46]. The BTACT scoring consist of two subscales episodic memory and executive functioning and their sum to estimate global functioning,all three scores were converted to z-scores to enhance interpretation [47, 48]. The BTACT was created to address the gap in telephone-based tests and is sensitive to normal cognitive functioning for adults without neurocognitive diseases such as dementia while allowing for greater access for individuals for whom in-person testing is not ideal [46]. Higher scores indicated better functioning, while − 1 to − 2 z-score could indicate middle impairment and less than − 2 suggests more significant impairment.

#### Ethnoracial Status

Participants were asked to endorse their ethnoracial origin(s) and afforded the opportunity to provide up to three among the following: White, Black and/or African American, Native American or Alaskan Native, Asian, Native Hawaiian or Pacific Islander, Other, or Don’t Know. Any endorsement of a non-White response across the three variables was coded by their first ethnoracial minority indicated. In addition, a question asked participants whether or not they identified as having Spanish/Hispanic/Latino descent. Due to small sample sizes in other non-White identities, the ethnoracial responses were coded as 0 = non-Hispanic White identity and 1 = any ethnoracial minority identity for regression analyses.

#### Allostatic Load

We constructed an estimate of multi-system dysregulation as a proxy for the accumulation of biological and physiological responses during everyday life; similar to past analyses with this dataset, allostatic load is connected to overall poor general well-being [40]. This allostatic load score is the sum of 26 biomarker values at M2. Dysregulation scores for biomarkers falling into the categories representing the cardiovascular (e.g., blood pressure), metabolic (e.g., cholesterol), neuroendocrine (e.g., urine catecholamines), and immune (e.g., C-reactive protein) systems were calculated using established clinical cutoff with 0 indicating individuals being within normal range and values up to 1 expressing levels of dysregulation. Other systems without well-established clinical cutoffs were calculated using the highest risk quartile of its distribution, accounting for the inverse association between several biomarkers (e.g., resting heart rate variability, grip strength) and suboptimal health outcomes.

#### Covariates

Demographic (age, gender, and education) as well as medication use, both all medications (sum of self-reported medications used) and antidepressant use (0 = no; 1 = yes) only, and allostatic load variables from M2 that could influence the association between depressive symptoms and cognitive functioning were controlled for in the analyses.

### Analytic Plan

Statistical analyses were conducted using the PROCESS Macro (version 4.1) and SPSS Statistics software (version 28). All tests are two-tailed and set at a significance level of $\alpha$ = 0.05. Age, sex, allostatic load, all medication use (except antidepressant use), antidepressant medication use, and education level were control variables for analyses including the whole sample. Sensitivity analyses were conducted by removing participants who endorsed antidepressant use at M2; all covariates were retained except for antidepressant use for the sensitivity analyses. Except for sex, ethnoracial minority status, and antidepressant medication use, all variables were analyzed as continuous. All continuous predictor variables were automatically mean-centered using the PROCESS macro prior to analysis to aid results interpretation and generate estimates to examine the interactions [49]. The moderating effect of ethnoracial minority status on the relationship between depressive symptoms and cognitive function was tested using PROCESS model 1. M2 and M3 cognitive functioning (2 subscales and overall) were examined separately, and M2 cognitive functioning was used as a control in the M3 analysis to estimate the change in cognitive functioning over time. All primary analyses were conducted with and without those who indicated using antidepressants at M2, given the known confound of major depression and cognitive functioning.

## Results

### Sample Characteristics and Key Variable Correlations

Table 2 presents the zero-order correlations among primary and confounding factors. As expected, M2 and M3 cognitive function were highly associated, *r* (739) = 0.79, *p* < 0.001. In addition, poorer M2 and M3 cognitive functioning were related to identifying as an ethnoracial minority, greater allostatic load, total number of medications used, elevated depressive symptoms, and older age, while higher M2 and M3 cognitive functioning were linked to greater education. The ethnoracial minority group was on average younger, had less education, more likely to be female, reported less medication use including antidepressant use, and endorsed greater depressive symptoms compared to their White counterparts. Please see Table 1 for more details.

**Table 2:** Zero-order correlations among primary and secondary study variables for the overall sample

Variables	1	2	3	4	5	6	7	8	9
1. M2 ethnoracial minority (yes)	–-								
2. M2 sex (female)	.09^**^	–-							
3. M2 education	− .15^***^	− .06^†^	–-						
4. M2 total medications	− .09^**^	.14^***^	.04	–-					
5. M2 antidepressant use (yes)	− .08^*^	.07^*^	.08^*^	.26^***^	–-				
6. M2 allostatic load	.02	− .04	− .11^**^	.21^***^	.16^***^	–-			
7. M2 age	− .08^*^	− .04	− .06^†^	.37^***^	− .01	.34^***^	–-		
8. M2 depressive symptoms	.11^**^	.03	− .11^**^	.05	.19^***^	.06^†^	− .14^***^	–-	
9. M2 global cognition	− .27^***^	.03	.38^***^	− .16^***^	− .06^†^	− .24^***^	− .36^***^	− .14^***^	–-
10. M3 global cognition^a^	− .25^***^	.01	.39^***^	− .13^***^	− .05	− .25^***^	− .38^***^	− .12^***^	.79^***^

*n* = 910, unless otherwise noted. ^a^*n* = 741. ^*†*^*p* < .10, *p < .05, **p < *.01*, ***p < *.001. M2*, Midlife in the United States wave 2 data collection; *M3*, Midlife in the United States wave 3 data collection

In this community-dwelling population that included those who were able to travel for an in person 2-day lab visit, 802 (88.1%) at M2 and 702 (94.7%) participants had global cognitive functioning in the normal range. Similar to the overall ethnoracial differences in M2 and M3 cognition, the ethnoracial minority group was likely to have more individuals whose scores indicated mild to significant impairment compared to their non-Hispanic White counterparts at M2 (46 [26.3%] vs. 62 [8.4%], respectively; *X*^2^ = 54.28, *p* < 0.001) and M3 (20 [14.6%] vs 19 [3.1%], respectively; *X*^2^ = 29.38, *p* < 0.001).

Individuals who reported using antidepressants at M2 were more educated (non-users = 7.4 ± 2.5 and users = 8.0 ± 2.5, *p* < 0.05), more likely to be non-Hispanic White (*X*^2^ = 5.5, *p* < 0.05), and identify as female (*X*^2^ = 4.8, *p* < 0.05) compared to non-users. As expected, those on antidepressants reported greater depressive symptoms (mean = 12.4 ± 10.5) compared to non-users (mean = 8.3 ± 7.1), *p* < 0.001, regardless of ethnoracial minority group, suggesting those who were taking antidepressants may have sought treatment for elevated depressive symptoms. Furthermore, antidepressant users, on average, reported using 2.5 more medications and had an elevated allostatic load compared to non-users (*p*’s < 0.05). While M2 cognitive functioning was marginally lower among antidepressants users (user =  − 0.01 ± 0.84 vs non-user = 0.15 ± 0.93, *p* = 0.06), the two groups did not differ on age or M3 cognitive functioning.

### Concurrent Relationship Between Depressive Symptoms and Cognitive Functioning

**Fig. 1:**
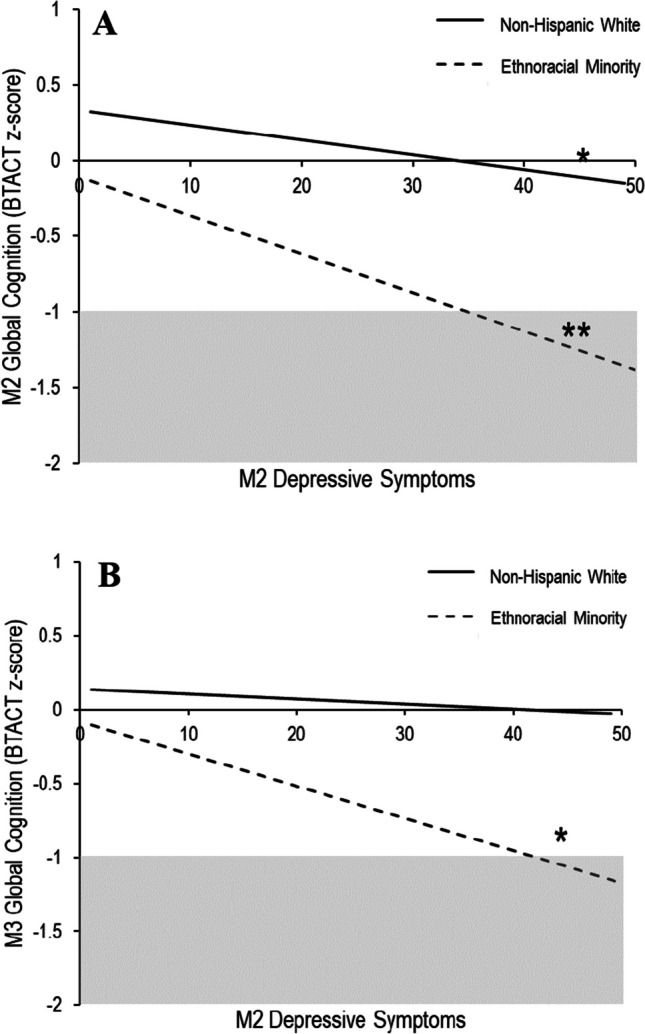
Simple slopes showing interaction among M2 depressive symptoms and ethnoracial minority group on cognition concurrently (**A**) and longitudinally (**B**). *Note.* *p < .05, **p < .001*.* M2, Midlife in the United States wave 2 data collection; M3, Midlife in the United States wave 3 data collection; BTACT, Brief Test of Adult Cognition by Telephone. Using PROCESS macro model 1, the two-way interaction between depressive symptoms and ethnoracial minority group significantly predicted cognitive functioning concurrently (**A**) and longitudinally (**B**) when controlling for sex, age, education, number of medications, antidepressant use, and allostatic load (*p*’s < .05). In **A**, both lines indicate a significant negative relationship; however, the relationship is stronger among the ethnoracial minority group (*β* =  − .026, *p* < .001) compared to the non-Hispanic white group (*β* =  − .009, *p* = .012). The longitudinal analysis also controlled for baseline or M2 cognitive functioning; thus, **B** provides the estimated change in cognitive functioning from M2 to M3. For the line representing ethnoracial minority group, M2 depressive symptoms predicted cognitive decline (*β* =  − .008, *p* = .044), while in the non-Hispanic white group, the line’s slope was not significantly different than 0. The gray box indicates BTACT z-scores in the mild cognitively impaired range

For the whole sample, depressive symptoms and ethnoracial minority status had significant main effects on overall cognitive functioning (see Table 3). Further, their interaction significantly predicted concurrent global cognition (Δ*R*^2^ = 0.003, *F* (1, 900) = 4.80, *p* = 0.029). Simple slopes revealed that for both non-Hispanic White participants and ethnoracial minorities, as depressive symptoms increased, cognitive functioning was poorer (see Fig. 1A). This relationship was stronger in ethnoracial minorities (*β*=  − 0.026, *p* < 0.001, 95% CI − 0.039, − 0.013) compared to non-Hispanic White participants (*β* =  − 0.009, *p* = 0.012, 95% CI − 0.017, − 0.002). The analyses among those not using antidepressant medication replicated the results of the whole sample; however, the interaction accounted for more variance overall (Δ*R*^2^ = 0.005, *F* (1, 760) = 5.48, *p* = 0.020).

**Table 3:** Summary of the analyses examining the interaction (PROCESS macro model 1) between depressive symptoms and ethnoracial minority status on global cognition concurrently and longitudinally with and without those who reported using antidepressants

Variable	M2 global cognition				M3 global cognition			
	All (*n* = 910)		Non-AD users (*n* = 769)		All (*n* = 741)		Non-AD users (*n* = 635)	
	*β*	[LLCI, ULCI]	*β*	[LLCI, ULCI]	*β*	[LLCI, ULCI]	*β*	[LLCI, ULCI]
1. M2 sex (female)	− .13^*^	[− .23, − .03]	− .14^*^	[− .25, − .03]	− .01	[− .07, .05]	.00	[− .06, .06]
2. M2 age	− .03^***^	[− .03, − .02]	− .03^***^	[− .03, − .02]	− .01^***^	[− .01, − .01]	− .01^***^	[− .01, − .01]
3. M2 education	.12^***^	[.10, .14]	.12^***^	[.10, .15]	.03^***^	[.02, .04]	.04^***^	[.02, .05]
4. M2 allostatic load	− .02^*^	[− .03, − .00]	− .01	[− .03, .00]	− .01	[− .02, .00]	− .01	[− .02, .00]
5. M2 total meds	− .01	[− .02, .00]	− .01	[− .02, .01]	.00	[− .01, .01]	− .00	[− .01, .01]
6. M2 AD use (yes)	− .20^**^	[− .34, − .06]	–-	–-	− .06	[− .15, .02]	–-	–-
7. M2 global cognition	–-	–-	–-	–-	.48^***^	[.45, .52]	.47^***^	[.43, .51]
6. M2 ethnoracial minority (ErM)	− .57^***^	[− .69, − .44]	− .57^***^	[− .71, − .43]	− .10^*^	[− .18, − .02]	− .11^**^	[− .19, − .03]
7. M2 depressive (Dep.) symptoms	− .01^*^	[− .02, − .00]	− .01^*^	[− .02, − .00]	.00	[− .00, .01]	.00	[− .00, .01]
8. ErM x Dep. symptoms	− .02^*^	[− .03, − .00]	− .02^*^	[− .04, − .00]	− .01^*^	[− .02, − .00]	− .01^*^	[− .02, − .00]
Interaction Δ*R*^*2*^ =	.003^*^		.005^*^		.002^*^		.003*	

^*^p < .05, ^**^p < .01, ^***^p < .001. *M2*, Midlife in the United States wave 2 data collection; *M3*, Midlife in the United States wave 3 data collection; *AD*, antidepressant; *β* beta coefficient; *LLCI*, lower limit of 95% confidence interval; *ULCI*, upper limit of 95% confidence interval; *Meds*, medications

For the subscale analyses, executive functioning mirrored the global cognitive functioning results, while episodic memory did not. Depressive symptoms and ethnoracial minority status had significant main effects on executive functioning (see Table 4). Further, their interaction predicted concurrent executive functioning at trend level (Δ*R*^2^ = 0.002, *F* (1, 900) = 3.07, *p* = 0.080). The analyses among those not using antidepressant medication replicated the results of the whole sample; however, the interaction accounted for more variance overall (Δ*R*^2^ = 0.004, *F* (1, 760) = 5.20, *p* = 0.023). Depressive symptoms, ethnoracial minority status, and their interaction were not predictive of episodic memory (all *p*’s > 0.189). The episodic memory analysis without antidepressant users was slightly different (see Table 5); ethnoracial minority status did significantly predict episodic memory at M2 (*β* =  − 0.230, *p* = 0.001, 95% CI − 0.374, − 0.086), but neither depressive symptoms nor the interaction predicted M2 episodic memory (all *p*’s > 0.296).

**Table 4:** Summary of the analyses examining the interaction (PROCESS macro model 1) between depressive symptoms and ethnoracial minority status on executive functioning concurrently and longitudinally with and without those who reported using antidepressants

	M2 executive functioning				M3 executive functioning			
	All (*n* = 910)		Non-AD users (*n* = 769)		All (*n* = 741)		Non-AD users (*n* = 635)	
Variable	*β*	[LLCI, ULCI]	*β*	[LLCI, ULCI]	*β*	[LLCI, ULCI]	*β*	[LLCI, ULCI]
1. M2 Sex (female)	.06	[− .04, .16]	.04	[− .06, .14]	− .01	[− .07, .05]	.07*	[.00, .14]
2. M2 Age	− .03^***^	[− .03, − .02]	− .03^***^	[− .03, − .02]	− .01^***^	[− .01, − .01]	− .01^***^	[− .02, − .01]
3. M2 Education	.11^***^	[.09, .13]	.11^***^	[.09, .14]	.03^***^	[.02, .04]	.03^***^	[.02, .05]
4. M2 Allostatic Load	− .01	[− .03, .01]	− .01	[− .03, .01]	− .01	[− .02, .00]	− .01	[− .02, .00]
5. M2 Total Meds	− .01^†^	[− .02, .00]	− .01	[− .02, .01]	.00	[− .01, .01]	.00	[− .01, .01]
6. M2 AD Use (yes)	− .20^**^	[− .34, − .06]	–-	–-	− .06	[− .15, .02]	–-	–-
7. M2 Executive Functioning	–-	–-	–-	–-	.48^***^	[.45, .52]	.50^***^	[.46, .55]
6. M2 Ethnoracial Minority (ErM)	− .48^***^	[− .67, − .29]	− .59^***^	[− .72, − .46]	− .02	[− .18, − .02]	− .09^*^	[− .18, − .00]
7. M2 Depressive (Dep.) Symptoms	− .01^*^	[− .01, − .00]	− .01^*^	[− .02, − .00]	.00	[− .00, .01]	.00	[− .00, .01]
8. ErM x Dep. Symptoms	− .01^†^	[− .03, .00]	− .02^*^	[− .04, − .00]	− .01^*^	[− .02, − .00]	− .01^*^	[− .02, − .00]
Interaction Δ*R*^*2*^ =	.002^†^		.004^*^		.002^*^		.003*	

^†^*p* < .10, ^*^p < .05, ^**^p < .01, ^***^p < .001. *M2*, Midlife in the United States wave 2 data collection; *M3*, Midlife in the United States wave 3 data collection; *AD*, antidepressant; *β* beta coefficient; *LLCI*, lower limit of 95% confidence interval; *ULCI*, upper limit of 95% confidence interval; *Meds*, medications

**Table 5:** Summary of the analyses examining the interaction (PROCESS macro model 1) between depressive symptoms and ethnoracial minority status on episodic memory concurrently and longitudinally with and without those who reported using antidepressants

Variable	M2 episodic memory				M3 episodic memory			
	All (*n* = 910)		Non-AD users (*n* = 769)		All (*n* = 741)		Non-AD users (*n* = 635)	
	*β*	[LLCI, ULCI]	*β*	[LLCI, ULCI]	*β*	[LLCI, ULCI]	*β*	[LLCI, ULCI]
1. M2 sex (female)	− .59^***^	[− .69, − .48]	− .54^***^	[− .65, − .42]	− .43^***^	[− .55, .30]	− .44^***^	[− .57, − .31]
2. M2 age	− .02^***^	[− .03, − .01]	− .02^***^	[− .03, − .01]	− .02^***^	[− .02, − .01]	− .02^***^	[− .02, − .01]
3. M2 education	.06^***^	[.04, .094]	.07^***^	[.04, .09]	.04^***^	[.01, .06]	.04^***^	[.01, .06]
4. M2 allostatic load	− .01	[− .03, .00]	− .01	[− .03, .01]	.01	[− .01, .03]	− .00	[− .02, .02]
5. M2 total meds	.00	[− .01, .01]	.00	[− .01, .02]	− .01	[− .02, .00]	− .01	[− .02, .01]
6. M2 AD use (yes)	− .12	[− .27, .04]	–-	–-	− .23^**^	[− .40, − .06]	–-	–-
7. M2 episodic memory	–-	–-	–-	–-	.45^***^	[.39, .52]	.46^***^	[.38, .54]
6. M2 ethnoracial minority (ErM)	− .10	[− .31, .11]	− .23^**^	[− .37, − .09]	− .04	[− .27, .18]	− .10	[− .26, .06]
7. M2 depressive (Dep.) symptoms	− .00	[− .01, .00]	− .00	[− .01, .01]	− .00	[− .01, .00]	.00	[− .01, .01]
8. ErM x Dep. symptoms	− .01	[− .03, .01]	− .01	[− .03, .01]	− .01	[− .02, .01]	− .01	[− .03, .01]
Interaction Δ*R*^*2*^ =	.001		.001		.001		.002	

^*^p < .05, ^**^p < .01, ^***^p < .001. *M2*, Midlife in the United States wave 2 data collection; *M3*, Midlife in the United States wave 3 data collection; *AD*, antidepressant; *β* beta coefficient; *LLCI*, lower limit of 95% confidence interval; *ULCI*, upper limit of 95% confidence interval; *Meds*, medications

### Depressive Symptoms Predict Cognitive Decline

For the whole sample, while controlling for M2 cognitive functioning, ethnoracial minority status, but not depressive symptoms, predicted cognitive functioning at M3—in other words cognitive decline at M3 from M2. The interaction reached significance (Δ*R*^2^ = 0.002, *F*(1, 730) = 4.08, *p* = 0.044). Specifically, depressive symptoms at M2 predicted greater cognitive decline for the ethnoracial minority group (*β* =  − 0.008, *p* = 0.044, 95% CI − 0.016, − 0.000]), but not for their non-Hispanic White counterparts (*β* = 0.001, *p* = 0.657, 95% CI − 0.003, 0.005). Similar to the concurrent analyses, the analyses among those not using antidepressant medication replicated the results of the whole sample and the model was stronger (Δ*R*^2^ = 0.003, *F* (1, 625) = 5.11, *p* = 0.024). See Table 3 for the detailed statistical model summaries and Fig. 1B for the graphical representation.

For the subscale analyses, executive functioning mirrored the global cognitive functioning results, while episodic memory did not. Neither M2 depressive symptoms nor ethnoracial minority status had significant main effects on the change in executive functioning (see Table 4). However, their interaction predicted M3 executive functioning (Δ*R*^2^ = 0.002, *F* (1, 769) = 4.26, *p* = 0.039). Specifically, depressive symptoms at M2 predicted greater decline in executive functioning for the ethnoracial minority group (*β* =  − 0.009, *p* = 0.029, 95% CI − 0.017, − 0.001), but not for their non-Hispanic White counterparts (*β* = 0.001, *p* = 0.814, 95% CI − 0.004, 0.005). The analyses among those not using antidepressant medication replicated the results of the whole sample (Δ*R*^2^ = 0.002, *F* (1, 769) = 4.26, *p* = 0.039). Depressive symptoms at M2, ethnoracial minority status, and their interaction were not predictive of M3 episodic memory (all *p*’s > 0.259). The analyses among those not using antidepressant medication replicated the results of the whole sample (all *p*’s > 0.166).

## Discussion

This study investigated the relationship between depressive symptoms and cognitive functioning concurrently and longitudinally in community-dwelling midlife adults and whether ethnoracial minority status altered that relationship. In line with prior literature, increased depressive symptoms were associated cross-sectionally with poorer cognitive functioning, regardless of ethnoracial minority status [7, 50]. In the longitudinal analysis, depressive symptoms predicted cognitive decline a decade later, but only among the ethnoracial minority group. The removal of antidepressant users strengthened the results, suggesting that antidepressants may alter the relationship between depressive symptoms and cognition functioning during midlife adulthood. Furthermore, these results held when controlling for key factors such as education and proxies for chronic health status such as medication use and allostatic load.

While prior longitudinal evidence suggests that depressive symptoms predict cognitive decline among primarily White populations [13–15], our findings suggest among middle to late midlife adulthood, depressive symptoms take a toll on the cognitive functioning among ethnoracial minority individuals rather than White individuals. This finding supports previous research that depression in ethnoracial minorities is associated with greater cognitive decline [16]. Additionally, the nonuniform predictive nature of depressive symptoms on cognitive function suggests that other factors are at play when considering this relationship [35], highlighting the need for further investigations into the role of sociodemographic factors and other group disparities on cognitive health and may suggest a differential presentation of depressive symptoms across ethnoracial groups [51].

The effect of depression on cognition appears wide-ranging and likely heterogeneous across sociodemographic characteristics [35]. For example, compared to previous longitudinal studies [13–15], the present sample is 10–20 years younger at baseline. These data suggest that the ethnoracial minority subsample may be experiencing greater negative repercussions due to depressive symptoms on cognitive functioning at an earlier age on average compared to their White counterparts. This interpretation is consistent with the cumulative advantage and disadvantage (CAD) theory [35]. Unfortunately, the observation of accelerated aging among ethnoracial minorities in the US is not unique to cognitive functioning; in fact, ethnoracial minorities develop chronic physical health conditions at an earlier age and have a shorter life expectancy compared to their White counterparts (e.g., [52, 53]). Thus, more cumulative harm throughout life may perpetuate the acceleration of cognitive aging that appears to be occurring within the ethnoracial minority group in this study.

Using a more inclusive approach, we conducted our analyses with all eligible participants, regardless of possible major depressive disorder. However, the removal of those on antidepressants led to an improved or strengthening of the models. M2 data were collected in 2004–2009 at a time when antidepressant use was primarily limited to those with moderate to severe depression, unlike today where antidepressants can be prescribed off label for a wide list of chronic health conditions such as obsessive–compulsive disorder, irritable bowel syndrome, migraines, and eating disorders [54–56]. Thus, the relationship between depressive symptoms and cognitive functioning may differ for those with major depressive disorder compared to a non-clinical sample. Our findings also suggest different rates of treatment of depression by ethnoracial minority status especially pharmacological treatment, which is consistent with prior research and may hint at the mistrust of the minority population in the healthcare system [16] or cultural attitudes which limit the use of modern medical practices [57]. Further research should attempt to replicate our findings in both clinical and non-clinical ethnoracially diverse populations.

Congruent with existing research, our results indicate that executive functioning seems to be a key area of disturbance, especially longitudinally, possibly driving the overall negative trend in cognitive function among individuals with depressive symptoms, especially those of the ethnoracial minority group [7]. Researchers posit that cognitive impairments in depression are dependent on age and the number of depressive episodes an individual has undergone [50, 58]. However, our results do not indicate a significant decline in the episodic memory score either concurrently or longitudinally, once again highlighting the inconsistent pattern of cognitive impairments and decline as a result of depressive symptoms [7, 50].

While our findings provide evidence for the long-established negative association between depression and cognitive functioning, the exact mechanism(s) has long eluded researchers [59]. Unfortunately, the cognitive battery used in this study was limited in its scope, precluding investigation into how specific cognitive domains are differentially impacted by depression [7]. Studies have investigated regression-based norms for the BTACT, however, due to the limited ethnoracial diversity of participants, ethnoracial-based norms have not been developed [47, 60]. Furthermore, the BTACT may have limited utility outside of screening for traumatic brain injury as its neuropsychological psychometric performance compared to more lengthy and comprehensive assessments is poor [61]. While these negatives may be explained by the exclusion of a visual component and difficulty to standardize due to possible distraction outside of laboratory settings [47], our findings provide insight into the multi-factorial role depressive symptoms may have on cognitive functioning among mid- and late life, community dwelling adults.

While we specifically examined the role of ethnoracial minority status, we were limited in our ability to examine differences among ethnoracial groups. The ethnoracial minority sample was a significantly smaller proportion of the overall sample (~ 19%) and predominantly Black/African American. Even the Black/African American category does not adequately capture the diversity within that ethnoracial background because the country of origin, SES, and educational backgrounds can vary within the Black/African American community [62]. Another limitation in our study was that depressive symptoms were only assessed at one timepoint, limiting a nuanced examination of how depressive symptoms may have fluctuated over the 10-year period. Similarly, the lack of M3 depressive symptoms does not allow for analysis of the directionality of the relationship between cognitive decline and depressive symptoms.

Further research should focus on investigating areas of cognitive impairment among depressed individuals and the possibility that these vary by ethnoracial identity [35]. The potential different presentation of depression across ethnoracial groups would serve as an essential piece in understanding the varying effect of depression [18]. Additionally, research into closing the treatment gaps and investigating the comorbidity of other chronic diseases, especially among the ethnoracial minority population, may contribute to a greater understanding of the outsized impact of depression among minority populations [18].

While previous research independently investigated depression [50] and ethnoracial minority status on cognition [16, 35], this study presents a novel contribution by examining the effect of ethnoracial minority status on the depression-cognitive functioning relationship both concurrently and longitudinally during mid- to late midlife. Based on our findings, ethnoracial minorities are at greater risk of depression-related cognitive decline at an early age compared to non-Hispanic white individuals. Depressive symptoms are considered modifiable (e.g., [63]), suggesting that identifying protocols to screen and treat depressive symptoms in midlife may improve cognitive functioning and quality of life as one ages.

## Data Availability

Data were retrieved from the Inter-university Consortium for Political and Social Reserach (ICPSR) and are publicly available for download. Data from Milwaukee substudy require an additional data sharing agreement that was signed and managed by Jeanette M. Bennett, PhD, with support from UNC Charlotte.
